# Evaluating Knowledge, Attitudes, and Behaviors toward HPV Infection and Vaccination among University Students in Italy

**DOI:** 10.3390/vaccines11101517

**Published:** 2023-09-24

**Authors:** Gabriella Di Giuseppe, Silvia Angelillo, Aida Bianco, Francesca Gallè, Francesca Licata, Giorgio Liguori, Francesco Napolitano, Carmelo Giuseppe Angelo Nobile, Maria Pavia, Concetta Paola Pelullo, Italo Francesco Angelillo

**Affiliations:** 1 Department of Experimental Medicine, University of Campania “Luigi Vanvitelli”, 80138 Naples, Italy; 2 Department of Health Sciences, University of Catanzaro “Magna Graecia”, 88100 Catanzaro, Italy; 3 Department of Movement Sciences and Wellbeing, University of Naples “Parthenope”, 80133 Naples, Italy; 4 Department of Pharmacy, Health and Nutritional Sciences, University of Calabria, Arcavacata of Rende, 87036 Cosenza, Italy

**Keywords:** attitudes, HPV, Italy, knowledge, survey, university students, vaccine, willingness

## Abstract

Background: This survey investigated the knowledge, attitudes, and behaviors towards HPV preventive measures among university students in Italy and their associated factors. Methods: The cross-sectional survey was conducted between November 2022 and April 2023. Results: Only 41.7% knew about HPV infection and the main preventive measures. Multilevel mixed-effects logistic regression analysis showed that females, those aged 25–30 years, those who have had oral sex, those who had received information about HPV infection and preventive measures from physicians, and those who needed additional information about HPV infection and preventive measures were more knowledgeable. Only 39.1% have received at least one HPV vaccination dose, whereas 29.2% and 31.7% had not been vaccinated or did not remember. Multilevel mixed-effects logistic regression analysis identified that female students enrolled in the field of health sciences, those who did not report a prior history of a sexually transmitted infection, and those with a higher knowledge about HPV infection and preventive measures were more willing to receive the HPV vaccination. Conclusions: Despite some limitations, this was the first detailed survey on this topic conducted in the post-acute phase of COVID-19 among university students in Italy. The survey underlined the need to develop and to implement comprehensive educational programs and health interventions among students, to enhance their knowledge and modify their attitudes and improve the HPV vaccine uptake.

## 1. Introduction

Human Papillomavirus (HPV) vaccines are the most effective method, along with other precautionary measures, to reduce the incidence of infections and prevent the associated cervical, vulvar, vaginal, oropharyngeal, anal, and penile cancers [1]. In Italy, the HPV vaccine is recommended and offered free of charge to adolescent males at 12 years and to females aged 12–25 years under the National Immunization Program [2]. Despite these recommendations, the vaccination coverage still lags behind the target of 95%, with only 32.2% and 26.7% female and male adolescents at 12 years having completed the vaccine series in 2021 [3].

In line with the initiation of the vaccination against HPV, a body of literature has focused attention on the level of knowledge, attitudes, and behaviors regarding preventive measures and the vaccination. When looking at this literature, many of the studies have been conducted among adolescents [4,5], parents [6,7,8], general population [9,10,11], and health care professionals [12,13,14]. During the COVID-19 pandemic, a worldwide decline in HPV and other vaccination coverage has been observed [15,16,17]. This is a major worry. However, it may be expected that the concern about the pandemic may be a positive key determinant for self-protection and intention towards HPV or other vaccinations. However, a limited number of studies have been conducted recently regarding HPV among university students. It is pivotal to address this knowledge gap since they are expected to be a sexually active population and, therefore, at higher risk of exposure to HPV. It is particularly important to conduct these surveys for the development and implementation of public health education campaigns in a way that is suitable for many individuals to either participate in a healthy lifestyle behavior, or to increase the vaccination uptake. Thus, this is the first survey of its kind in the post-acute phase of COVID-19 and the purposes were to investigate the level of knowledge of HPV infection and of the main preventive measures, the attitudes regarding the risk of acquiring an HPV infection, the behavior towards HPV preventive measures, and the willingness to receive HPV vaccination among university students in Italy and to understand the various associated factors.

## 2. Materials and Methods

### 2.1. Study Design, Participants, and Sampling Procedures

This survey is part of larger research activities that investigated the genotype-specific prevalence of HPV infection in southern Italy’s young population in urine and oral samples. A cross-sectional survey was conducted between November 2022 and April 2023. The target population consisted of students enrolled in a degree program in the fields of medicine, healthcare professions, pharmacy, and sports science from three public universities in various geographic locations of the southern part of Italy. In particular, the sample was selected with a two-stage cluster method. In the first stage, 25 degree courses have been selected from the lists of the three universities and in the second stage, from each course the students have been selected through a simple random sampling. The sample size of the survey was determined to be 768, assuming an expected genital HPV prevalence of 20% in the non-vaccinated population and a post-vaccination HPV 16/18 prevalence of 10%, with 95% confidence intervals and a 0.05 margin of error, and a design effect of 2. In addition, accounting for an expected response rate of 90%, the minimum number was increased to 853. Overall, a total of 1652 students were selected.

### 2.2. Data Collection and Questionnaire Design

The members of the research team approached the students on randomly selected days during their courses. A self-administered questionnaire was used for data collection. It required approximately 15 min to answer all the questions. At the beginning of the questionnaire, the students were informed of the main objectives of the survey, assured that it was completely anonymous, that the participation was voluntary, that the responses would be treated with complete confidentiality, that they could withdraw at any time during questionnaire completion without consequences, that they would not receive any financial compensation, that the data would be used for the objectives of the survey, and that the informed consent for their participation was implied by returning the questionnaire.

Six pieces of information were collected in the questionnaire. In the first part, all participants completed questions exploring socio-demographic and general characteristics including gender, age, and family history of cancer. In the second part, ten items assessed the level of knowledge about HPV. Five of these questions were closed-ended with “Yes”, “No”, and “Do not know” answers and the other five were multiple-choice types and participants were allowed to choose more than one answer from a list of the options offered. In the third part, concerns about the perception of the risk of acquiring an HPV infection or a related disease, or a sexually transmitted disease, were assessed with three items and for these questions the responses were measured on a five-point Likert scale with 1 being “strongly disagree/not at all” and 5 being “strongly agree/very much”. Three questions about preventive measures were asked including the perceived utility of the vaccination, of the Pap test, and of the DNA HPV test. In the fourth part, participants indicated whether they had been vaccinated against HPV and those who were unvaccinated responded to a question exploring their willingness or uncertainty or unwillingness to take it, and also to indicate the most likely reason(s) for their willingness or uncertainty or unwillingness from five options. The last part included questions on the main sources of HPV-infection and related preventive measures information and participants were requested to tick any of the five options offered. Finally, they were asked whether additional information was needed.

Before the questionnaire was distributed, a pilot study was conducted among a small group of 50 students in the field of health sciences to ensure the questionnaire was easy to understand and to answer.

### 2.3. Statistical Analysis

STATA software version 17 was used for the statistical analysis. First, the different information was presented as mean and standard deviation for the continuous variables and frequency and proportion for the categorical variables. Second, univariate analysis using the Chi-square test or Student’s t-test was performed to assess the associations, respectively, between categorical and continuous variables and the outcomes of interest. Third, independent variables with a *p*-value ≤ 0.25 in the univariate analysis were included in three multilevel mixed-effects logistic regression models to calculate the odds ratio (OR) and the 95% confidence interval (CI) in order to verify the significant relationship between dependent variables and independent variables. With the aim of accounting for the multilevel dataset structure (procedures are “nested” within degree courses), the variable degree courses were introduced in the models as a random factor. The outcomes of interest were the following: knowledge about the HPV infection and the preventive measures (score 0–4 = 0; score 5–10 = 1) (Model 1); quite/very concerned about the risk of acquiring an HPV infection (not at all/slightly/neutral = 0; quite/very = 1) (Model 2); and willingness to receive the HPV vaccination (no = 0; yes = 1) (Model 3). The outcome of Model 1 assessed the level of knowledge about HPV with the ten questions and it was created by giving the respondent a score of “1” for each correct answer and “0” for each incorrect or unknown answer. The total knowledge score for each respondent was calculated and ranged from 0 to 10; those with a score above the median value of 4 was considered to be knowledgeable, and coded with “1”, and those with a score below or equal to 4 to be unknowledgeable, and coded with “0”. The following independent variables have been tested for all outcomes: gender (male = 0; female = 1), age (18–24 = 0; 25–30 = 1), undergraduate health sciences course (no = 0; yes = 1), sexual orientation (asexual/bisexual/gay/lesbian/pansexual = 0; heterosexual = 1), family history of HPV-related cancers (no = 1; yes = 2; other = 3), having smoked at least 100 cigarettes during lifetime (no = 0; yes = 1), consuming alcohol (no = 0; yes = 1), having had sexual intercourse during lifetime (no = 0; yes = 1), current partners (none = 1; casual = 2; regular = 3), having had oral sex during lifetime (no = 0; yes = 1), having had a diagnosis of a sexually transmitted infection (STI) (no = 0; yes = 1), physicians as source of information about HPV infection and preventive measures (no = 0; yes = 1), and needing additional information about HPV infection and prevention (no = 0; yes = 1). The variable HPV vaccination status (no = 0; yes = 1; unknown = 2) was included in Models 1 and 2 and knowledge of HPV infection and preventive measures (score 0–4 = 0; score 5–10 = 1) in Models 2 and 3. To assess the level of knowledge, an overall knowledge score was constructed giving 1 point for each correct answer to the ten questions about HPV and respondents with a score above the median were considered to have knowledge of HPV infection and of the main preventive measures. Multiple imputation (10 imputed datasets), by assuming that data were missing at random, with chained equations with linear regression for continuous variables and logistic regression for binary variables has been used to fill the missing values. All variables from the logistic regression analysis have been included in the imputation models. To perform multiple imputation analysis, the multilevel mixed-effects logistic regression models have been fitted to the multiple imputed datasets and the results combined using Rubin’s rules. For all analyses, two-sided tests, *p*-values ≤ 0.05, were considered statistically significant. The results from the multiple imputation analysis have been presented as the primary study results.

## 3. Results

Of the 1652 randomly selected students, 1612 returned the questionnaire for a response rate of 97.6%. The principal characteristics of the enrolled participants are described in Table 1. The mean age of the participants was 21.9 years, more than half were female and were medical students, almost all were heterosexual, 35% had smoked at least 100 cigarettes during their lifetime, 87.6% consumed alcohol, more than three-quarters have had a sexual intercourse and half of them currently had a regular partner, less than three-quarters often/always used condoms during their sexual activity, 73.3% have had oral sex, and only 5.3% had a family history of HPV-related cancers.

Table 2 reports the answers of the ten questions that were used to assess the respondents’ knowledge about HPV. The vast majority were able to recognize HPV as a sexually transmitted infection (88%), that it interested both sexes (90.2%), and that it is responsible for cervical cancer (78.4%), although the other associated cancers have been identified by a lower number of respondents ranging from 33.2% for the oral to 51% for the penile. The efficacy of the HPV vaccine in preventing cervical cancer was recognized by two-thirds of the sample (66%) and only 7.6% for oral and penile cancers. The majority indicated that condom use helps to prevent the transmission (80%), but only 20% indicated a lower number of sexual partners.

The median value of the knowledge score was 4 (range = 0–10) and less than half of the participants (41.7%) had a score greater than or equal to 5 and were knowledgeable about HPV infection and the main preventive measures. Multilevel mixed-effect logistic regression analysis was used to assess which of the several characteristics influenced the different outcomes of interest and the results for both the complete cases and multiple imputed data are shown in Table 3. The first model was performed to identify factors associated with the level of knowledge about HPV infection and preventive measures. Females (OR = 1.4; 95% CI = 1.06–1.84), those aged 25–30 years (OR = 2.07; 95% CI = 1.47–2.9), those who have had oral sex (OR = 1.52; 95% CI = 1.08-2.15), those who had received information about HPV infection and preventive measures from physicians (OR = 1.77; 95% CI = 1.33–2.35), and those who needed additional information about HPV infection and preventive measures (OR = 1.87; 95% CI = 1.47–2.32) were more likely to be knowledgeable about HPV (Model 1).

A multilevel mixed-effect logistic regression model was performed to identify factors associated with the level of knowledge about HPV among those vaccinated and the results showed that participants aged 25–30 years (OR = 3.12; 95% CI = 1.74–5.39), those who had received information about HPV infection and preventive measures from physicians (OR = 1.52; 95% CI = 1.01–2.26), and those who needed additional information about HPV infection and preventive measures (OR = 1.85; 95% CI = 1.3–2.58) were more likely to be knowledgeable about HPV.

The statements of the perceived risk of acquiring an HPV infection or an HPV-related cancer or a sexually transmitted disease, measured on a 5-point Likert-type scale, indicated that, respectively, 23.9%, 50.7%, and 45.3% were quite/very concerned. The multivariate logistic regression analysis revealed that three factors predicted participant’s perceived risk of HPV. Females (OR = 1.54; 95% CI = 1.13–2.09), those knowledgeable about HPV infection and of the main preventive measures (OR = 1.69; 95% CI = 1.31–2.18), and those who needed additional information about HPV infection and preventive measures (OR = 1.53; 95% CI = 1.19–1.97) were more likely to be quite/very concerned about the risk of acquiring an HPV infection (Model 2 in Table 3). A high level of perceived utility of the vaccine, of the Pap test, and of the DNA HPV test, measured on a 10-point Likert-type scale, has been observed with mean total values of 8.9, 8.8, and 8.4, respectively.

Only 39.1% of the respondents clearly remembered having received at least one HPV vaccination dose, whereas 29.2% and 31.7%, respectively, did not receive it or did not remember whether they had been vaccinated. Multivariate logistic regression analysis identified that females (OR = 2.29; 95% CI = 1.52–3.43), those enrolled in the field of health sciences (OR = 1.66; 95% CI = 1.11–2.48), those who did not report a prior history of a STI (OR = 0.3; 95% CI = 0.11–0.91), and those knowledgeable of HPV infection and of the main preventive measures (OR = 1.52; 95% CI = 1.02–2.26) were more willing to receive the HPV vaccination (Model 3 in Table 3). Regarding the most cited reasons behind why participants were willing to be vaccinated, 70.5% believed that the vaccine could reduce the risk of getting an HPV infection and 59% had high trust in the vaccination, whereas the top reason indicated for being unwilling to be vaccinated was not feeling at risk of infection (63.6%).

More than two-thirds of the sample had received information about HPV infection (67.9%) and related preventive measures (66.9%). Physicians and the Internet were mentioned as the two principal sources of information, with overall values of 22% and 20.3%, respectively. More than half (54.5%) of the students expressed the need to have additional information.

## 4. Discussion

This is the first detailed survey on the level of knowledge, attitudes, and behaviors towards HPV infection and preventive measures in the post-acute phase of COVID-19 among university students in Italy. Several implications for public health interventions and communication strategies can be drawn from this survey.

First, assessing the level of knowledge identified some major gaps among the sampled students for the different items regarding HPV. Indeed, only one-third and one-half were able to recognize, respectively, that oral and penile cancers were HPV-associated, two-thirds correctly identified the role of the HPV vaccine in preventing cervical cancer, and only 20.1% that a lower number of sexual partners helps to prevent HPV transmission. Given these gaps, improving the level of knowledge is of particular importance because the present survey reported that having a high level of general knowledge about HPV was positively associated with a favorable attitude towards vaccination and a higher likelihood of intention among the unvaccinated respondents who are at a higher risk.

Second, only 39.1% have already received at least one HPV vaccination dose, whereas 29.2% and 31.7% have not been vaccinated or did not remember. This figure is close to that from Switzerland where 36.9% of university students had received at least one HPV vaccination dose [18], whereas a remarkably higher value of 76.4% has been found among individuals aged 18 to 26 years who had received at least two doses in the US [19], and lower values of 21% in medical students in India [20], and 4.3% in undergraduate university students in Turkey [21]. The substantial differences that emerged in the prevalence across the studies could possibly be accounted for by variations in several factors, such as the year of the survey, sampling procedures, socio-demographic backgrounds of the studied population, health-care workers recommendation, difficulty accessing vaccination, parent’s attitudes, and the health-care delivery system. The very unsatisfactory vaccination uptake underscored the urgent need to implement targeted public health strategies and to dispense educational activities to both parents in order to vaccinate their children when they are eligible, and students in order to motivate and to encourage them to receive the HPV vaccine.

Third, the willingness to receive the HPV vaccine among those unvaccinated or who did not remember was very low with a value of 59.4%, whereas 33.9% were uncertain, and 6.7% did not want to be vaccinated. This observed intention was considerably lower than the values of 69.8% among female university students in Kuwait [22], and 65.2% in medical, dental, and nursing students in India [23]; whereas it was considerably higher than the 22.5% in university students in China [24] and the 4.6% in the already mentioned sample in the US [19]. It is worth noting that the low perceived risk of getting the HPV infection was the main reason for being unwilling to be vaccinated. Similar results have been observed in previous surveys among different groups of participants [4,25,26,27,28,29].

Fourth, the other findings of the multivariate logistic regression analysis indicated that several socio-demographic and general characteristics, such as age, gender, university course, sexual behaviors, and health status, were independently identified as significant determinants of the outcomes of interest. Understanding these characteristics is useful for identifying individuals for targeted efforts to increase, for example, the HPV vaccination uptake. This survey adds to the prevailing evidence that respondents aged 25–30 years had a higher level of knowledge. One possible explanation for these associations could be that those that are younger are less exposed to the infection risk and consequently they perceive themselves to be at lower risk while, in contrast, as age increases, the prevalence of the infection and of the associated diseases increases, making those that are older more knowledgeable, with a more positive attitude towards the importance of the vaccination. Moreover, knowledge about HPV infection and preventive measures, concern about acquiring an HPV infection and a related cancer, and willingness to receive the vaccine were observed to be significantly higher in women. In the literature, similar surveys from different countries among different groups of individuals have shown that women generally have a higher knowledge and more positive attitudes towards HPV vaccination than men [28,30,31]. The most reasonable explanations for the differences observed according to gender, are that this vaccination is recommended and offered free of charge to healthy females at the age of 12–25 years and to males only at 12 years, and that health education activities to prevent HPV infection have focused primarily on cervical cancer. Therefore, male students may be less aware of the potential impact of HPV infection on them and on the benefits of the vaccination. However, the low level of knowledge about HPV in men increases the risk of transmission. This finding has been observed in similar surveys conducted worldwide [29,32]. Another interesting observation, in agreement with previous studies from different countries [33,34], was that students enrolled in the field of health sciences are more willing to receive the vaccination compared to those in a non-health-related field. This finding was expected and could be explained by the fact that students in health-related fields receive information on HPV infection, associated cancers, and preventive measures through their courses, compared to those in non-health-related fields. These findings further emphasize the importance of adequate health information that may play an important role in educating students in non-health-related fields. Not surprisingly, students who have had oral sex reported a higher knowledge. This may be because those who are engaged in sexual behaviors with a substantial risk of getting an HPV infection have more interested in the vaccination. In addition, participants who did not report a prior history of an STI were almost 70% more likely to be willing to receive the HPV vaccination than those with a prior STI diagnosis.

Fifth, assessments of the principal sources used to gain information about HPV infection and related preventive measures, revealed that physicians, although only in about a fifth of the respondents, were the most widely used, followed by the internet. The findings regarding the sources of information clearly provide evidence and offer insight that physicians may directly and positively impact respondents’ knowledge and attitudes. Indeed, respondents who received information from physicians had higher HPV infection and preventive measures knowledge and positive health care attitudes. Numerous studies among diverse populations from different geographic areas, have found that healthcare professionals, especially physicians, are the preferred source of health-related information and have emphasized the importance of providing preventive campaigns, which may translate into safer and more appropriate behaviors [35,36]. However, of great concern is that only about a fifth of the respondents had received information from physicians, considering that they are an important source that can positively reflect healthier life habits in the population of university students. Furthermore, most respondents stated the need for more information, and this is a salient theme, particularly because in the multivariate logistic regression analysis, this need was significantly associated with all but one outcome of interest. Thus, more interaction with physicians, mainly those involved in direct and longer interactions with patients and their families, and tailored educational interventions are required to provide accurate information on the risk factors and on the benefits of the preventive measures and to support and to facilitate decision-making processes among this population.

## 5. Survey Limitations

It is necessary to acknowledge the possibility of some methodological limitations when interpreting the findings. First, the cross-sectional nature of this survey limits the ability to attribute causation to the associations that have been observed between the independent variables and the outcomes of interest. Second, the population sample was selected from two geographic areas in the Southern part of Italy and, therefore, caution is needed in generalizing these findings more broadly to all Italian students. Third, the data were gathered through a self-administered questionnaire and therefore, the recall bias of participants may have influenced the results, for example, on the sexual habits and on immunization status. Fourth, participants might have more positively answered the questions and therefore, for example, due to the sensitive nature of those regarding sexual behaviors, might be subject to social desirability bias. However, keeping the identity of the participants anonymous, we are confident that this limitation was likely to have had little influence on the results.

## 6. Survey Implications

The results highlight the need to increase students’ knowledge on HPV. This should be attempted with educational interventions, by involving both schools and universities and physicians, and also by taking advantage of other opportunities to access health services such as primary care visits and vaccination centers. Moreover, the HPV vaccination coverage and the willingness to receive it were very low. Therefore, there is ample room for healthcare policymakers and managers to implement appropriate preventive strategies. Efforts should be made to increase vaccination coverage through targeted interventions that provide a more accessible service, increase the trust in the HPV vaccine for parents and young people, and involve healthcare professionals in preventive programs.

## 7. Conclusions

In conclusion, the present survey underlined that it is needed to develop and to implement comprehensive educational programs and health interventions as essential tools to meet the information needs of this population and that will contribute to enhancing their knowledge, modify their attitudes and improve the HPV vaccine uptake. The findings of this survey are also useful for future investigations focusing on attitudes and behaviors of healthcare professionals towards HPV infection and vaccination given their key role in increasing vaccination coverage.

## Figures and Tables

**Table 1:** Main characteristics and sexual behaviors of the respondents.

	Total (*n* = 1612)		*Having Received the HPV Vaccination*			
			Yes (*n* = 630)		No/Do Not Remember (*n* = 982)	
Characteristics	N	%	N	%	N	%
*Age*	21.9 ± 2.4 (18–30) *		21.7 ± 2.1 (18–30) *		22 ± 2.6 (18–30) *	
18–24	1368	86.7	557	40.7	811	59.3
25–30	210	13.3	68	32.4	142	67.6
*Gender*						
Female	974	60.4	388	39.8	586	60.2
Male	638	39.6	44	6.9	594	93.1
*Sexual orientation*						
Heterosexual	1499	93.9	581	38.8	918	61.2
Asexual/bisexual/gay/lesbian/pansexual	97	6.1	45	46.4	52	53.6
*Undergraduate course*						
Medical area	851	52.8	429	50.4	422	49.6
Other	761	47.2	560	73.6	201	26.4
*Having smoked at least 100 cigarettes during lifetime*						
No	1047	65	634	60.5	413	39.5
Yes	565	35	217	38.4	348	61.6
*Consuming alcohol*						
No	200	12.4	60	30	140	70
Yes	1412	87.6	570	40.4	842	59.6
*Having had sexual intercourse during lifetime*						
No	365	22.6	154	42.2	211	57.8
Yes	1247	77.4	476	38.2	771	61.8
*Current sexual partners*						
Regular	863	65.3	358	41.5	505	58.5
Casual	145	11	43	29.7	102	70.3
None	313	23.7	104	33.2	209	66.8
*Lifetime gender of sexual partners*						
Opposite	1129	92.4	426	37.7	703	62.3
Same	55	4.5	22	40	33	60
Both	38	3.1	19	50	19	50
*Condom use during sexual intercourse*						
Never/rarely/sometimes	466	37.4	195	41.8	271	58.2
Often/always	781	62.6	281	36	500	64
*Having had oral sex during lifetime*						
No	431	26.7	172	39.9	259	60.1
Yes	1181	73.3	458	38.8	723	61.2
*Condom use during oral sex*						
Never/rarely/sometimes	1077	91.2	419	38.9	658	61.1
Often/always	104	8.8	39	37.5	65	62.5
*Family history of HPV-related cancers*						
No	779	48.3	287	36.8	492	63.2
Other cancers	748	46.4	313	41.8	435	58.2
Yes	85	5.3	30	35.3	55	64.7
*Having received at least one HPV vaccination dose*						
No	471	29.2				
Do not remember	511	31.7				
Yes	630	39.1				

* Mean ± Standard deviation (range). Number for each item may not add up to total number of study population due to missing values.

**Table 2:** Knowledge about HPV of the respondents.

			*Having Received the HPV Vaccination*			
	Total (*n* = 1612)		Yes (*n* = 630)		No/Do Not Remember (*n* = 982)	
	N	%	N	%	N	%
*HPV is a sexually transmitted infection*						
Yes	1418	88	582	41	836	59
No	194	12	48	24.7	146	75.3
*HPV infection can affect*						
Females	37	2.3	14	37.8	23	62.2
Males	33	2	10	30.3	23	69.7
Females and males	1454	90.2	589	40.5	865	59.5
Do not know	88	5.5	17	19.3	71	80.7
*HPV related cancers*						
Cervical	1263	78.4	543	43	720	57
Penile	823	51	318	38.6	505	61.4
Anal	591	36.7	221	37.4	370	62.6
Oral	535	33.2	231	43.2	304	56.8
*Preventive measures for HPV infection*						
Condoms (yes)	1292	80.2	510	39.5	782	60.5
Reducing number of sexual partners (yes)	324	20.1	118	36.4	206	63.6
Oral disinfectants (no)	69	4.3	27	39.1	42	60.9
Contraceptive (no)	62	3.8	16	25.8	46	74.2
*HPV vaccination prevents*						
Cervical cancer	1073	66.6	476	44.4	597	55.6
Genital cancer	593	36.8	182	30.7	411	69.3
Anal cancer	122	7.6	45	36.9	77	63.1
Oral cancer	122	7.6	46	37.7	76	62.3
*A pap-test is an effective screening method for early detection of cervical cancer*						
Yes	1241	77	537	43.3	704	56.7
No/Do not know	371	23	93	25.1	278	74.9
*DNA testing for HPV is an effective screening method for early detection of cervical cancer*						
Yes	827	51.3	313	37.8	514	62.2
No/Do not know	785	48.7	317	40.4	468	59.6
*Urine test for early detection of genital cancer*						
Yes	603	37.4	236	39.1	367	60.9
No/Do not know	1009	62.6	394	39	615	61
*Urine test for early detection of cervical cancer*						
Yes	525	37.6	176	33.5	349	66.5
No/Do not know	1087	62.4	454	41.8	633	59.2
*Saliva test for early detection of oral cancer*						
Yes	400	24.8	122	30.5	278	69.5
No/Do not know	1212	75.2	508	41.9	704	58.1

**Table 3:** Multilevel mixed-effect logistic regression analysis results examining the outcomes of interest according to several explanatory variables on both the complete cases and multiple imputed data.

	Complete Cases				Multiple Imputation			
Variable	OR	SE	95% CI	*p* Value	OR	SE	95% CI	*p* Value
Model 1. Knowledge of HPV infection and of the main preventive measures								
Aged 25–30 years	2.12	0.36	1.52–2.97	<0.001	2.07	0.36	1.47–2.9	<0.001
Needing additional information about HPV infection and preventive measures	1.87	0.22	1.49–2.35	<0.001	1.87	0.21	1.47–2.32	<0.001
Physicians as source of information about HPV infection and preventive measures	1.75	0.26	1.32–2.33	<0.001	1.77	0.26	1.33–2.35	<0.001
Females	1.39	0.19	1.05–1.83	0.02	1.4	0.19	1.06–1.84	0.016
Having had oral sex	1.52	0.27	1.07–2.16	0.018	1.52	0.27	1.08–2.15	0.017
*HPV vaccination status*								
Yes	1 *				1 *			
Do not remember	0.73	0.11	0.54–0.99	0.05	0.74	0.11	0.54–1.01	0.051
No	1.23	0.19	0.91–1.67	0.173	1.24	0.19	0.92–1.67	0.16
Reporting a prior history of a sexually transmitted infection	1.67	0.66	0.77–3.63	0.192	1.72	0.68	0.79–3.73	0.168
Students enrolled in the field of health sciences	1.49	0.63	0.65–3.42	0.343	1.54	0.64	0.68–3.49	0.297
*Current sexual partners*								
None	1 *							
Regular	1.08	0.14	0.83–1.41	0.562	1.04	0.14	0.81–1.35	0.767
Casual	0.74	0.17	0.47–1.15	0.181	0.69	0.15	0.44–1.07	0.095
Consuming alcohol	1.12	0.2	0.78–1.59	0.538	1.07	0.19	0.76–1.54	0.644
Being a smoker	1.07	0.13	0.84–1.36	0.581	1.08	0.13	0.85–1.36	0.537
*Family history of HPV-related cancers*								
Yes	1 *							
No	0.87	0.22	0.53–1.43	0.592	0.88	0.22	0.54–1.44	0.614
Other cancers	1.06	0.27	0.65–1.75	0.804	1.09	0.27	0.66–1.78	0.742
Having had sexual intercourse during lifetime	1.03	0.2	0.71–1.51	0.88				
Model 2. Quite/very concerned about the risk of acquiring an HPV infection								
Knowledge of HPV infection and of the main preventive measures	1.63	0.21	1.26–2.11	<0.001	1.69	0.22	1.31–2.18	<0.001
Needing additional information about HPV infection and preventive measures	1.59	0.21	1.21–2.01	0.001	1.53	0.19	1.19–1.97	0.001
Females	1.49	0.23	1.09–2.03	0.01	1.54	0.24	1.13–2.09	0.005
*Family history of HPV-related cancers*								
Yes	1 *							
No	0.59	0.16	0.35–1.01	0.053	0.61	0.16	0.36–1.02	0.058
Other cancers	0.8	0.21	0.48–1.34	0.395	0.81	0.21	0.48–1.35	0.407
Reporting a prior history of a sexually transmitted infection	1.85	0.72	0.87–3.96	0.112	1.88	0.73	0.88–4.04	0.103
Asexual/bisexual/gay/lesbian/pansexual	0.72	0.17	0.45–1.16	0.182	0.71	0.17	0.45–1.15	0.164
Physicians as source of information about HPV infection and preventive measures	1.19	0.19	0.88–1.62	0.264	1.2	0.19	0.88–1.63	0.247
Students enrolled in the field of health sciences	1.38	0.47	0.71–2.71	0.340	1.4	0.19	0.71–2.76	0.324
Having had oral sex	1.07	0.16	0.81–1.43	0.632	1.09	0.16	0.82–1.45	0.554
*HPV vaccination status*								
Yes	1 *							
Do not remember	0.89	0.15	0.63–1.25	0.511	0.91	0.16	0.65–1.28	0.607
No	1.02	0.17	0.73–1.42	0.899	1.02	0.17	0.74–1.42	0.896
Aged 25–30 years	1.04	0.19	0.73–1.51	0.817	1.04	0.19	0.72–1.51	0.820
Model 3. Willingness to receive HPV vaccine								
Females	2.26	0.51	1.45–3.51	<0.001	2.29	0.47	1.52–3.43	<0.001
Knowledge of HPV infection and of the main preventive measures	1.54	0.34	1.01–2.36	0.047	1.52	0.31	1.02–2.26	0.039
Students enrolled in the field of health sciences	2.23	0.91	1.01–4.98	0.049	1.66	0.34	1.11–2.48	0.013
Not reporting a prior history of a sexually transmitted infection	0.37	0.21	0.11–1.16	0.088	0.3	0.17	0.11–0.91	0.034
Asexual/bisexual/gay/lesbian/pansexual	0.51	0.24	0.21–1.31	0.164	0.41	0.19	0.16–1.01	0.052
Physicians as source of information about HPV infection and preventive measures	1.24	0.34	0.73–2.13	0.422	1.22	0.31	0.74–2.01	0.443

* Reference category.

## Data Availability

The data presented in this study are available upon request from the corresponding author.
